# A Comprehensive Assessment of Knowledge, Attitudes, and Practicalities Related to Doping Agents use among Jordanians

**DOI:** 10.2174/17450179-v18-e2202280

**Published:** 2022-03-25

**Authors:** Mohanad Odeh, Haneen M. Tailakh, Abdel Qader F. Al Bawab, Nour A. Elsahoryi, Karem H. Alzoubi

**Affiliations:** 1 Department of Clinical Pharmacy and Pharmacy Practice, The Hashemite University, Zarqa, Jordan; 2 Department of Pharmacy, Faculty of Pharmacy, Al-Zaytoonah University of Jordan, Amman, Jordan; 3 Department of Nutrition, Faculty of Pharmacy and Medical Sciences, The University of Petra, Amman, Jordan; 4 Department of Pharmacy Practice and Pharmacotherapeutics, College of Pharmacy, University of Sharjah, Sharjah, UAE; 5 Department of Clinical Pharmacy, Jordan University of Science and Technology, Irbid, Jordan

**Keywords:** Doping Agents, Knowledge, Attitudes, Practices, Anabolic-androgenic steroids (AAS), Jordan

## Abstract

**Background:**

People perform sports for better health and wellbeing. However, the use of doping agents is emerging among young adults. This study investigated aspects related to doping agents.

**Methods:**

A reliable self-administered questionnaire (Cronbach’s alpha =0.72, Pearson's r = 0.89) was used to assess knowledge, attitudes, and practicalities related to the use of doping agents. Results for pharmacists as health care providers (HCP, n=550) were compared with non-healthcare providers (Non-HCP, n=319).

**Results:**

Among pharmacists, 82.9% knew the definition of doping agents *vs*. 72.4% of non-HCP (P<0.001). However, 36.7% of pharmacists *vs*. 39.6% of non-HCP incorrectly classified doping agents (P=0.02). The majority of responders (89.8%) supported having an anti-doping authority, yet, only 15% were aware of the anti-doping organizations. The majority of responders (83%) did not receive an official education related to doping agents. Enhancing physical performance was perceived as a leading driver (82.1%) to use doping agents. More than 90% of responders supported awareness in the community. The perceived best tool for awareness was social media and TV sites, as suggested by pharmacists (95.0%) and non-HCP (92.1%, P=0.312). A total of 6.1% had ever used doping agents (3.6% pharmacist *vs*. 9.8% non-HCP, P<0.001). Almost half of the users utilized a diet or medication to counteract the side effects of doping agents. Within pharmacists, males received more requests to provide doping agents (41.9%) compared with females (23.8%, P<0.001).

**Conclusion:**

It is crucial to enhance professional and legal knowledge and public awareness about doping agents, not only for non-HCP but also for HCPs. Applying more restrictions on doping agents is strongly recommended.

## 1 INTRODUCTION

Exercise is vital for human health and wellbeing. It is associated with better health outcomes and enhanced muscle profile [1]. Introducing externally abnormal materials as a supposed performance-enhancing substance (PES) will lead to abnormal adaptation in the body as well as long-term negative consequences. Such methods and practices are mostly illegal and unethical practices [2, 3]. Risks are magnified if such PES are used with inadequate medical supervision.

The World Anti-Doping Agency (WADA) was established in 1999 [4]. It is a self-governed international association aimed toward bringing consistency to anti-doping policies and regulations within sports organizations and governments across the world [5]. In 2000, the Olympic Movement Anti-Doping Code was established. It defined doping in two ways: an abstract definition and a “pragmatic” definition based on the restricted list of substances [6]. Article number two of the code states that “doping is the use of an expedient (substance or method) which is potentially harmful to athletes' health and/or capable of enhancing their performance. Pragmatically, it is the presence of a prohibited substance or evidence of the use thereof or evidence of the use of a prohibited method in the athlete's body” [2].

Despite being perceived as beneficial in the short-term response, doping causes serious health adverse events, including liver, heart, vascular diseases, severely impacts sexual functions, and leads to behaviour changes and mood disturbances. Moreover, practicing irresponsible doping and using medical substances without medical supervision was associated with sudden death [2, 3].

Doping is a widespread practice in the young population. There is a belief that doping is a useful way for better achievement in sports, either for competitive or non-competitive exercise training. It is more common in bodybuilding and weight lifting sports [2, 3, 7]. Yet, several studies disagreed with the safe and effective use of anabolic-androgenic steroids (AAS) in improving muscle mass and strength performance [8-11]. For example, Andrews and colleagues in their systematic review, assessed muscular strength, body composition, cardiovascular endurance, and power. They found a small absolute increase in strength attributed to AAS and a moderate increase in lean mass. Nevertheless, they confirmed that the adverse effects of AAS were not rigorously assessed nor reliably reported [8]. D Van Gammeren *et al*. and F Hartgens *et al*. showed no change in muscle fibers, body composition, circumference measures, strength, force, power, and exercise performance with the use of AAS [9, 10]. Another very recent review has listed an excellent summary of studies between 2006–2019 that documented adverse effects of anabolic steroid use on the brain and behaviour, as well as body systems, including the cardiovascular, urinary, musculoskeletal, and reproductive systems, the liver, and the blood [12].

Unfortunately, using doping agents has not always been considered cheating [13]. Athletes may be persuaded to use steroids due to misleading themes such as “winning at all costs” and “dying to win,” in addition to perceived cost-benefit [14]. Moreover, the sports coaches' attitudes toward doping showed that 70% believed that doping is a common reason to break sports records and that no doping will minimize the opportunity to achieve success [15, 16]. In all cases, it is challenging to prevent young adults and even older athletes from using doping medications to boost their performance, even if medical and scientific evidence exists [17], as dopers were considered potential addicts that need addiction treatment [18].

Multiple studies and systematic reviews were carried out to explore broad aspects of sports medicine, doping agents, and anti-doping techniques [19]. Researchers consistently investigated clinical, humanistic, pedagogic, behavioural, and legal aspects related to doping and subsequent substances [17, 18, 20-25]. In particular, a number of studies have investigated knowledge, attitude, and beliefs about doping in sports [13-16, 18, 26]. The focus of most researchers was related to anabolic hormones. However, there is a common agreement that knowledge, correct classification of substances, and awareness about doping are still insufficient and poorly evaluated, even among pharmacists [27].

Doping-related science is poorly addressed in Jordanian curricula, and the qualification competency requirements for health care providers (HCPs), such as physicians, pharmacists, and nurses, are inadequate [2, 4, 28, 29]. Unfortunately, a lack of professional and proper knowledge leads to misleading rumours and myths. For example, illegal promotion and the circulated rumours minimise the awareness about doping adverse events, which results in more consumption of such agents. Accordingly, this study aimed to carry out a comprehensive investigation about doping agents among Jordanian pharmacists as HCP versus a population of non-healthcare providers (non-HCP). A validated and reliable questionnaire was used to assess details related to knowledge, attitudes, and practicalities.

## 2 METHODS

The study was carried out through an online self-administered questionnaire, which was organized to assess the following knowledge: basic definition (2 questions), doping agents' proper classification (15 questions), basic education and source of information (4 questions), legal knowledge (6 questions), and side effects’ knowledge (6 questions). The second part assessed the following attitudes: personal attitude toward doping (2 questions), attitudes toward users (2 questions), penalties (3 questions), attitude toward social awareness (4 questions), perceptions about benefits (3 questions), and drivers for doping (5 questions). The third part investigated practicalities among users of doping agents such as prevalence, regimen to counteract side effects, and source of providing the agents. Additional practicalities among pharmacists were related to quantifying requests for providing doping agents and readiness to report issues related to doping agent users.

### 2.1 Tool Development

The basic questionnaire was designed to assess knowledge, attitude, awareness approaches, and other aspects related to doping agents. The authors created a panel of specialist pharmacists and non-healthcare providers who practice work related to sports and coaching. This panel enhanced the process of questionnaire validation (face and criterion validity). The panel members were as follows: assistant professors in the following disciplines: pharmacotherapy, pharmaceutics, pharmacologists, clinical pharmacists; a pharmacist who is a board member in the Jordanian Pharmacist Association, a pharmacy director of the hospital pharmacy department, and a physician. Non-HCPs associated with sport-related issues were as follows: a director of accounting, an accountant and part-time coach, an engineer, an administrative director, a personnel trainer, and a sports consultant. All panel members agreed on the final form of the questionnaire with its suggested criterion scoring.

Reliability [27, 28] was confirmed by two means: the internal consistency measured by Cronbach’s alpha value and the stability over time (*i.e*., test-retest reliability). A pilot sample of 40 responders was contacted twice within a 15 days time difference. They were asked to answer the questionnaire to carry out the pre-/post-tests. Overall internal consistency was calculated using the coefficient alpha (Cronbach’s alpha was 0.72), and test-retest reliability was assessed using Pearson’s correlation coefficient (Pearson's *r* was 0.89). The coefficient alpha results reflect reliable acceptable internal consistency. The results of the stability coefficient indicated stronger test-retest reliability. Accordingly, the measurement error of the questionnaire was less likely to be attributable to changes in the individuals’ responses over time [30, 31].

### 2.2 Sample Size Calculation

Jordanians aged between 15-64 years were 6,532,966 (*i.e*., 61% of Jordanian population) as reported by the Department of Statistics of Jordan as per the census of 2019 [32]. Accordingly, a representative sample size with a Confidence Level (CI) of 95% and 5% marginal error was calculated to be 385 participants, as recommended by Taherdoost [33] and carried out by the sample size calculator [34]. Furthermore, a subsample to represent pharmacists was calculated to be 381 participants, with 95% CI and 5% margin error for a population of no more than 40,000 pharmacists [35].

### 2.3 Sample Collection

This study used a cross-sectional design using Google form templates to distribute the survey, which was active for receiving answers between 1^st^ Dec 2019 till 30^th^ April 2020. The study protocol was approved by the Hashemite University IRB committee on the 25th of Nov, 2019 (No.9 – Code: 9/25/11/2019). Eligible responders were those pharmacists and non-healthcare providers who would like to voluntarily respond to the survey. The survey link was distributed using social media platforms (Facebook) and WhatsApp applications through the study panel members. Responders were informed that their responses would be treated confidentially and anonymously. There was no personal identifier in the survey. Responders were also informed that they had the choice to withdraw from the survey, as their participation is voluntary. Accordingly, participants who submitted the survey with their answers were considered to have given informed consent for their participation.

### 2.4 The Statistical Analysis

A chi-square test of association was conducted between answers to the questions within the survey and speciality of responders, according to the following hypothesis:

The null hypothesis: H0: no association relationship between answers and the speciality (pharmacists *vs*. non-HCP).

The alternative hypothesis: HA: an association relationship exists between answers and the speciality (pharmacists *vs*. Non-HCP).

The measure of effect size, magnitude, and strength of association of a nominal-by-nominal relationship was assessed by Carmer V Coefficient, according to the following “crude estimates” for interpreting strengths of relationships.

## 3 RESULTS

### 3.1 General Description of Responders (Gender, Speciality, Income, Sport, Smoking)

Total responses were 919. The number of pharmacists who participated in the questionnaire was 550 (59.8%), and the number of non-HCP was 369 (40.2%). While male pharmacists dominated in non-HCP (239, 64.8 percent), female pharmacists dominated in HCP (340, 61.8 percent), resulting in a gender distribution of males and females that was comparable (48.9% males *vs*. 51.1% females). While 11.4% of male responders had a monthly income between 1001 and 2000 Jordanian Dinar (JD), less than 1% of females had such income. Almost 7% of males had a monthly income of more than 2000 compared with less than 0.06% of females. It was notable that almost half of the females, 49.1%, had no job, while for males, this proportion was 19.8%. 21% of both males and females reported having a monthly income of between 301 and 500 JD. Similarly, both have a proportion of almost 12% for the monthly income of less than 300 JD. Males within both pharmacists and non-HCP generally reported higher-income categories than females. However, no association was reported between income and the usage of doping agents.

Regarding practising sports, around 26% stated that they practice sports many times during the week, while almost 13% stated that they were poor sports performers (several times a year). Pharmacists and non-HCPs had almost similar proportions for participating in sports (X^2^ (5)=8.35, P=0.138). While 74.7% of pharmacists were non-smokers, the proportion was 50.7% for non-HCP. This difference was statistically significant (X^2^ (1) = 56.2, P <0.001).

### 3.2 Definition of Doping

Interestingly, the majority of the participants claimed to be knowledgeable about the topic of “Doping.” Doping agents were claimed to be known by both pharmacists and non-HCP. A higher proportion of pharmacists (80.9% *vs*. 74.3%, P=0.017) confirmed their knowledge about doping agents. A higher proportion of pharmacists compared with non-HCP (82.9% *vs*. 27.4%, P < 0.001) were familiar with the proper definition of doping agents.

### 3.3 Knowledge about Substances’ Classification of Agents and Medications

Poor knowledge about doping agents classification has been illustrated in the results of the present study. The proportions (pharmacists *vs*. non-HCP) who correctly identified the doping agent classes are shown in Table **1**. It was expected that smokers' *vs*. non-smokers' responses to the question of whether caffeine is a doping agent would be different. Nevertheless, the analysis showed no significant difference, as 39.8% of non-smokers *vs*. 34.6% of smokers thought that caffeine belongs to the doping agents (X^2^ (2) = 2.43, P=0.296, Cramer's V=0.051). On another front, a significantly higher proportion of females (62.2%) compared to males (37.8%) considered caffeine as a doping agent (X^2^ (2)=32.926, P<0.001, Cramer's V=0.189).

### 3.4 Knowledge about Side Effects

Pharmacists demonstrated significantly better knowledge about the side effects profile of the doping agents. A higher proportion of the pharmacists were able to identify the side effects of the doping agents compared with non-HCP; impotence (64.7% *vs*. 51.5%, P<0.001), heart and kidney diseases (74.7% *vs*. 62.9%, P<0.001), sudden death (70.9% *vs*. 56.9%, P<0.001), and acne (46.5% *vs*. 30.4%, P<0.001).

### 3.5 Source of Information and Education

The answers related to questions about formal education were almost similar in both study groups. Approximately 83% of both pharmacists and non-HCPs studied nothing about doping in school or university. Similar results were reported for receiving educational lectures about doping where almost 65% in both groups never received educational support nor read a scientific text about doping agents and their harmful effects. Compared to pharmacists (37.5%), significantly more non-HCPs (49.1%) thought that the sports coach provides the proper scientific guidance on doping to athletes (P<0.001).

### 3.6 Knowledge Related to the Legal Frame

According to the findings of this study, 84.9% (n=780) support the use of deterrent penalties to prohibit doping usage or trade. This shows a reliable construct with 79.1% (n=727) of responders who do not support the doping agents to become a legally permitted substance in Jordan. 89.8% (n=825) supported having an organization that prohibits doping agents and reduces their consumption.

Unfortunately, the majority (777, 84.5%) was not aware of the Jordanian Anti-Doping Organization (JADO), and 70.8% of responders (n=651) did not know about any organization that regulates sports anti-doping. Moreover, less than half (399, 43.4%) confirmed that the doping trade is a crime punishable by law. A similar proportion (407, 44.3%) confirmed the presence of Jordanian law to regulate doping. More details are shown in Table **2**.

### 3.7 Perceived Benefits of the Doping Agents

No differences were reported between both study groups in their attitude toward perceived benefits of doping agents for physical (79.3% *vs*. 81.3%, P=0.45) and mental performance (21.1% *vs*. 24.9%, P=0.17).

### 3.8 Perceptions about Reasons for the Use of Doping Agents

As seen in Table **3**, the most common perceived reason for the use of doping agents was to enhance physical performance (82.1%; agree = 316, 34.3%; strongly agree 438, 47.7%) followed by building body mass (80%; agree = 289, 31.4%; strongly agree 447, 48.6%). The weakest perceived driver was improving mental performance (34.9%; agree = 171, 18.6%; strongly agree 150, 16.3%). The distribution of answers between pharmacists and non-HCP was almost similar.

We decided to check the differences in the answers’ distribution based on gender. Against our expectations, data analysis showed that both females and males answered with equal proportions to the questions that explored drivers for doping agents’ usage among athletes. No significant differences were reported between both groups in their perception toward the consumption of doping agents for muscular enhancement, weight management, enhancing physical or mental performance, and copying with others. The only exception was at the sub-analysis with non-HCP females and males in the question about bodyweight management. In that question, a significantly higher (P<0.05) proportion of non-HCP males compared to females agreed that doping usage is for bodyweight management. Approximately 46% of non-HCP males agreed that maintaining body weight is the main reason for doping agents’ usage, while 31% of non-HCP disagreed (X^2^ (4) = 11.18, P=0.025, Cramer's V =0.175). A different trend was noticed in the pharmacists' group, where almost 32.3% of males agreed that maintaining body weight is the main reason for the usage of doping agents compared to 42.7% of females. Yet, the overall analysis showed that the proportions were not statistically significant within the pharmacists' group (X^2^ (4) = 6.73, P=0.151, Cramer's V =0.11).

### 3.9 Attitude Toward Doping Agent Users

Less than 10% supported the use of doping agents (5.6% of pharmacists *vs*. 8.9% of non-HCP, P=0.054) and considered it a moral act (6.2% of pharmacists *vs*. 8.45 of non-HCP, P=0.2). Pharmacists more than non-HCP supported the elimination of the doping agents user from sports competition (90.5% *vs*. 82.9%, P=0.001) and considered an individual liability for the use of doping agents (90.0% *vs*. 48.8%, P=0.018). Similarly, pharmacists more than non-HCP supported the application of deterrent penalties for doping agents users (87.3% *vs*. 81.3%, P=0.013).

### 3.10 Attitude toward Raising Community Awareness about Doping

More than 90% of both study groups showed a positive attitude toward creating awareness about the doping agents in the community. Moreover, 87.1% of responders shared responsibility and confirmed that they would like to educate others about the doping agents. Most (94.4%) of the participants supported conducting educational courses and lectures to create awareness about the names, types, and side effects (harms) of the doping agents. The majority (93.8%) supported creating awareness through social media and TV channels. The second-ranked awareness tool recommended by responders (90.8%) was the inclusion of lectures into official courses in universities and schools. Finally, the study participants (82.4%) supported posting brochures, adding road signs, and publishing newspaper articles to enhance awareness about doping agents.

### 3.11 Practicalities (Prevalence of Doping Agent users and Regimen to Counteract Side Effects)

As seen in Table **4**, a total of 6.1% (n=56) of respondents used doping agents before. However, non-HCP had a significantly higher proportion of doping agent use compared with pharmacists (9.8% *vs*. 3.6%, Carmer V = 0.125, P<0.001). A total of 31 out of 56 responders (55.4%) used a diet or drug after taking the doping agents to protect their bodies from side effects. Out of the 56 responders, nine responders did not specify any regimen, while 47 (83.9%) listed the regimen they used to counteract possible side effects, which were as follows: 19 (40.4%) used vitamins, 15 (31.9%) used a diet or natural mixtures, and 13 (27.7%) used a medication regimen such as Clomid^®^, Pregnyl^®^, Tamoxifen^®^. A dominant positive attitude toward such regimens was noticed across both groups. The vast majority (more than 90%) confirmed that they received positive information about such regimens and beneficial claims to reduce the side effects that might follow the doping agents' usage. However, less proportion (77.4%) recommended the use of such regimens to others. More than half (61.3%) did not use regimens to hide the lab test during official competitions. Almost one-third of the doping agents users obtained doping agents through their sports friends or sports coaches.

### 3.12 Practicalities with Pharmacists as Service Providers

Almost one-third of pharmacists received a request to obtain the doping agents for customers. Male pharmacists reported that they received significantly more requests than female pharmacists, P<0.001. Both males (84.3%) and females (88.8%) felt a high level of responsibility toward people's education. However, less proportion of both males (63.3%) and females (70.6%) felt a responsibility toward officially reporting doping dealers. Most responders agreed that the medical profession raises awareness about doping (91%). However, male pharmacists reported in higher percentage (6.7%) compared with females (2.1%) that awareness about the doping agents is not related to the medical profession. Results are shown in Table **5**.

## 4 DISCUSSION

### 4.1 Knowledge

In the present study, a gap between knowledge, perceptions, and practicalities was identified. Pharmacists, in the present study, claimed their knowledge about the doping agents; however, the in-depth assessment showed a gap in their knowledge. In a Japanese study, 82.2% of pharmacists claimed to be knowledgeable about doping agents [36]. In the present study, the best sub knowledge domain was the knowledge of side effects of the doping agents, where almost two-thirds of the participants reported that the doping agents can cause sexual weakness leading to sterility, and three-quarters of pharmacists reported that the doping agents cause cardiovascular and kidney diseases. These percentages were higher than those reported among German adolescents, where only 33.7% agreed that steroids cause kidney disease; however, 72.7% agreed that the doping agents are harmful [37]. In Saudi Arabia, the participants in a cross-sectional study agreed that the doping agents are considered dangerous for health (36.7%), treason (30.2%), and are against sportsmanship (33.1%) [38].

Remarkably, 82.9% of pharmacists confirmed that they were aware of the correct definition of the doping agents, while only 57% of Slovenian pharmacists and doctors reported in a similar assessment [4] that they did not know the correct definition of the doping agents. Such answers indicate a perception of good general knowledge in the Jordanian medical staff; however, it remains questionable, as in-depth knowledge of the doping agents and their classification showed a gap between self-assessed and actual knowledge.

### 4.2 Knowledge Classification

As the sample included pharmacists and non-pharmacists, the most common and local names, in addition to well-known scientific names, were used to identify the doping agents. Unfortunately, poor knowledge among pharmacists was demonstrated. A comparable level of knowledge with non-HCP was noticed, as well. In almost all questions, less than half of the pharmacists identified correct answers. The only exception was testosterone, where 71.3% of pharmacists had the correct answer. This is not unexpected, as testosterone is mentioned abundantly in medical books, especially those related to pharmacy, as it increases masculinity and shows a male appearance. Moreover, it was agreed previously that the main reason for using the doping agents is bodybuilding. This corresponds to a study of Slovenian pharmacists [4], where 92.8% rated testosterone as a doping agent. Contrary to our expectations, only 24% of pharmacists classified erythropoietin correctly as a doping agent. No statistically significant differences were found when comparing the answers of pharmacists with non-HCPs. This result came against that reported by Slovenian researchers [4], where 98.6% of their sample correctly classified erythropoietin as a doping agent. Classification of the following agents: ginseng, creatine, and phosphodiesterase (PDE) inhibitors as Sildenafil (Vigra^®^) and Tadalafil (Cialis^®^), has been assessed for the first time with the doping agents.

### 4.3 Knowledge about Effectiveness and Side Effects

Despite the strong evidence on both the short- and long-term side effects of AAS, there is very little evidence based on the users' perception of the negative consequences of its usage [12], especially among young adults and adolescents [37]. Pharmacists reported good awareness of side effects in both Qatar [28] and Japan [36]. In the present study, it was found that the first reason to use the doping agents was to enhance physical performance, followed by building body mass. This is in line with many studies, which reported that users aim to improve athletic performance and physical attractiveness, increase body weight, fat-free mass, muscle size, strength, and social recognition [2, 23, 26, 29, 38, 39]. Andrews *et al*., in their systematic review, listed a detailed reporting from 13 randomized trials about the adverse effects of AAS. The most commonly assessed adverse effects were negative impact on lipids, mood, and liver-associated enzymes [8]. Albano *et al*., in their very recent review, confirmed that the long-term administration of AAS may lead to serious consequences, such as hypogonadism, cardiac impairment, neurodegeneration, coronary artery disease, and sudden cardiac death [12].

### 4.4 Sources of Information and Education

Despite its prevalence and negative consequences, most of the responders did not study doping in schools and universities. A similar result was reported in France, where only 4% of the general practitioners reported having a specific class about doping [40]. Moreover, most responders neither read a scientific text nor attended an awareness lecture about doping. This is in line with results reported by Japanese pharmacists, where 83.5% did not receive any formal lecture about doping [36].

### 4.5 Legal Knowledge

Despite the attitude of prohibiting the doping agents, a high percentage of responders had poor knowledge about JADO. More than half of the participants believed there is no doping law in Jordan. The justification for this is straightforward: increased awareness of JADO and the doping-related laws is crucial. The legal knowledge about doping in Saudi Arabia seemed to be better than in Jordan, as 77.5% of the Saudi sample knew the punishment that followed doping abuse, and 67.6% considered it fair [41]. The phenomenon of awareness about doping agents is a global problem. It is not a unique case in Jordan. In Slovenia, only 55.3% of the community did not know about the anti-doping organization of their country [4]. In Qatar, none of the respondents were aware of the role of the World Anti-Doping Agency (WADA) [28].

### 4.6 Reasons for the use of Doping Agents

In the present study, most participants believed that the most common reasons for using the doping agents are to enhance physical performance, followed by building muscle mass. The results were somehow similar to those from Saudi Arabia, where 69.4% believed that the most common reason for doping was to enhance physical performance, whereas the second most common reason was social recognition (17%) [41]. Nevertheless, such results are contrary to the Syrian study's findings, where 77.9% indicated the most common reason for doping was building muscle mass, followed by enhancing physical performance (52.5%) [2].

### 4.7 Prevalence and Attitude toward the Doping Agents Users

In the present research, the overall prevalence of use of the doping agents was 6.1% (3.6% pharmacists and 9.8% non-HCP, P<0.001). Nevertheless, the real-life prevalence would be expected to be higher. This was a self-reported result; the academic endorsement of the questionnaire and ethical approval was announced. Thus, individuals may be reluctant to share information about the use of the doping agents to avoid liability and personal discomfort. It could be argued that respondents who took the doping agents (6.1%) are almost the same respondents who support taking such agents (7.0%) and consider it a moral action (7.1%). It is expected that they participate in sports competitions, where 7.8% of responders and 6.9% did not support eliminating the doping agent users from sports competitions, nor supporting a deterrent punishment for them. A similar trend was also noticed in another study carried out in Syria [2], where the proportion of responders who used the doping agents was almost equal to those considering their use as a moral act. However, the prevalence of the doping agent users in the present study is more than that reported in Syria (4.6%) and in Saudi Arabia (4.3%), but less than the prevalence in Germany (30.1%) [37]. The number of those who consider doping to be a moral act in the Syrian study was more than the number reported in the current study. This percentage reached 15.3% in the Syrian study [2]. In Japan, 10% considered doping as an acceptable violation [36], and in Qatar, 11% did not support doping to be prohibited [28].

### 4.8 Sources of the Doping Agents

In the present study, almost one-third of the doping agent users obtained them through their sports friends. The second-largest providers were sports coaches. Such results confirm the previous findings from Syria [2], Germany [37], and France [40].

### 4.9 Regimen to Counteract Adverse Events

Almost half of the doping agent users (55.4%) used a regimen to counteract possible side effects of those agents. The serious risk is that some regimens will increase long-term negative consequences. Medicines such as Clomid^®^, Pregnyl^®^, and Tamoxifen^®^ were used by one-third of the doping agent users. Such medicines increase female hormone levels where the rationale of such usage is to create balance with the exogenous anabolic (masculine) hormones. The unseen risk here is having very high levels of such hormones, more than the standard levels in the human body. On the other hand, using a healthy diet (rich in vitamins and natural products) would not be a scientifically validated method to protect against the harmful effects of the doping agents. Our assessment led us to say that such myths of using regimens to counteract side effects are circulated without scientific evidence. Our assessment showed that the most common source to sell such agents were sports coaches and sports friends. Accordingly, the scientific background of sports coaches and conflict of financial interest should be crucially considered by both individuals and authorities when dealing with such regimens.

### 4.10 Attitude toward Community Awareness about Doping

A positive attitude toward supporting community awareness was not unexpected. Similar results were reported from Slovenia [4]. In the present study, most of the responders supported applying deterrent penalties against the doping agents' abuse. Most responders did not want the doping agents to become legal. With consistent reliability, the vast majority (89.8%) supported the presence of an official Jordanian institution to prohibit doping and reduce its consumption.

### 4.11 Practicalities with Pharmacists as Service Providers

Although they felt less responsibility toward reporting claims against doping dealers, both males (84.3%) and females (88.8%) felt a high level of responsibility toward people's education. Such percentages were more than the Slovenian general practitioners and pharmacists (65%) [4] and the Syrian pharmacist (68.5%) [2]. An almost similar percentage was reported by French general practitioners, 89% [40]. About one-third of Jordanian medical staff had been exposed to a prescription containing a doping agent. A similar percentage received a request for providing an illegal doping agent. This is a high percentage when compared to Slovenian and French general practitioners, as their exposure to direct demand for prescribing steroids was 14% [4] and 11%, respectively [40].

## CONCLUSION

The present study makes several noteworthy contributions to the understanding of the aspects related to the doping agents among the population and the pharmacists as health care providers. To our knowledge, this is the first study that sheds light on such an important topic in Jordan. The study illustrated that the Jordanian population claimed reasonable knowledge about the doping agents. However, proper knowledge related to the classification and legal issues of the doping agents was poor. Athletes reported the usage of the doping agents despite their knowledge of their harmful consequences. There is a general perception that the doping agents enhance physical performance and build muscle mass. Although these facts are true, the misuse may lead to tragic scenarios. Many people reported using doping agents although they are not sports professionals and they do not aim to participate in sports competitions. This illustrates the general probability of using the doping agents despite their possible side effects. Unfortunately, myths about the negative sequences of the doping agents are circulated. It is worthy to emphasize that the resources for the doping agents need to be restricted and more regulated. Such action is expected to prevent doping malpractice. The evidence from this study reported that pharmacists did not receive proper knowledge regarding the doping agents. Therefore, doping-related issues should be included in the curricula of pharmacy programs. Regardless of the reasons, we strongly recommend investing more efforts to enhance public awareness and professional knowledge. Future studies are recommended to investigate in more depth the aspects of the doping agents in order to come out with stronger and more evidence-based conclusions.

## Figures and Tables

**Table 1:** Participants’ answers (Is the following agent a doping agent?).

**Question**	**Answer**	Pharmacist	Non- HCP	Total	X^2^	P	Carmer V	Correct Classification
Hormones (Deca, susta)	Do not belong	39	20	59	1.776	0.183	0.066	Doping agent
		7.1%	5.4%	6.4%				
	I do not know	255	261	516				
		46.4%	70.7%	56.1%				
	Belong	256	88	344				
		46.5%	23.8%	37.4%				
Hormones (Dianabol, Anabol)	Do not belong	28	22	50	4.43	0.035	0.113	Doping agent
		5.1%	6.0%	5.4%				
	I do not know	310	260	570				
		56.4%	70.5%	62.0%				
	Belong	212	87	299				
		38.5%	23.6%	32.5%				
Hormones (Anavar)	Do not belong	37	18	55	0.062	0.804	0.015	Doping agent
		6.7%	4.9%	6.0%				
	I do not know	366	285	651				
		66.5%	77.2%	70.8%				
	Belong	147	66	213				
		26.7%	17.9%	23.2%				
Male sex hormones (Testosterone)	Do not belong	51	49	100	23.443	<0.001	0.194	Doping agent
		9.3%	13.3%	10.9%				
	I do not know	107	189	296				
		19.5%	51.2%	32.2%				
	Belong	392	131	523				
		71.3%	35.5%	56.9%				
Growth hormones	Do not belong	101	65	166	4.17	0.041	0.81	Doping agent
		18.4%	17.6%	18.1%				
	I do not know	121	160	281				
		22.0%	43.4%	30.6%				
	Belong	328	144	472				
		59.6%	39.0%	51.4%				
Vitamins	Do not belong	247	151	398	2.119	0.145	0.054	Non-doping agent
		44.9%	40.9%	43.3%				
	I do not know	87	113	200				
		15.8%	30.6%	21.8%				
	Belong	216	105	321				
		39.3%	28.5%	34.9%				
Proteins (Amino acids)	Do not belong	186	91	277	0.190	0.663	0.018	Non-doping agent
		33.8%	24.7%	30.1%				
	I do not know	139	176	315				
		25.3%	47.7%	34.3%				
	Belong	225	102	327				
		40.9%	27.6%	35.6%				
Energy drinks	Do not belong	151	98	249	1.115	0.291	0.039	Non-doping agent
		27.5%	26.6%	27.1%				
	I do not know	85	99	184				
		15.5%	26.8%	20.0%				
	Belong	314	172	486				
		57.1%	46.6%	52.9%				
Ginseng	Do not belong	183	82	265	1.611	0.204	0.055	Non-doping agent
		33.3%	22.2%	28.8%				
	I do not know	173	219	392				
		31.5%	59.3%	42.7%				
	Belong	194	68	262				
		35.3%	18.4%	28.5%				
Caffeine	Do not belong	234	121	355	1.607	0.205	0.048	Non-doping agent
		42.5%	32.8%	38.6%				
	I do not know	102	113	215				
		18.5%	30.6%	23.4%				
	Belong	214	135	349				
		38.9%	36.6%	38.0%				
Cocaine	Do not belong	188	98	286	0.001	0.980	0.001	Doping agent
		34.2%	26.6%	31.1%				
	I do not know	171	171	342				
		31.1%	46.3%	37.2%				
	Belong	191	100	291				
		34.7%	27.1%	31.7%				
Marijuana	Do not belong	226	111	337	0.365	0.546	0.025	Doping agent
		41.1%	30.1%	36.7%				
	I do not know	178	178	356				
		32.4%	48.2%	38.7%				
	Belong	146	80	226				
		26.5%	21.7%	24.6%				
Creatine	Do not belong	205	109	314	0.004	0.947	0.003	Non- doping agent
		37.3%	29.5%	34.2%				
	I do not know	215	190	405				
		39.1%	51.5%	44.1%				
	Belong	130	70	200				
		23.6%	19.0%	21.8%				
Erectile dysfunction agents (Viagra, Cialis)	Do not belong	216	102	318	2.629	0.105	0.068	Non-doping agent
		39.3%	27.6%	34.6%				
	I do not know	178	169	347				
		32.4%	45.8%	37.8%				
	Belong	156	98	254				
		28.4%	26.6%	27.6%				
Erythropoietin	Do not belong	121	30	151	2.107	0.147	0.08	Doping agent
		22.0%	8.1%	16.4%				
	I do not know	297	291	588				
		54.0%	78.9%	64.0%				
	Belong	132	48	180				
		24.0%	13.0%	19.6%				

**Table 2:** Knowledge related to the legal frame for the doping agents.

**Question**	**Answer**	Pharmacist	Non HCP	Total	X^2^	P	Carmer V	P
Internationally prohibited								
There is a Jordanian organization to combat (prohibit) doping	Yes	168	100	268	1.269	0.26	0.037	0.26
		30.5%	27.1%	29.2%				
	No	46	56	102				
		8.4%	15.2%	11.1%				
	Not sure	336	213	549				
		61.1%	57.7%	59.7%				
Do you know what JADO abbreviation is?	Yes	83	56	139	0.001	0.972	0.001	0.972
		15.1%	15.2%	15.1%				
	No	398	271	669				
		72.4%	73.4%	72.8%				
	Not sure	69	42	111				
		12.5%	11.4%	12.1%				
Did you know that JADO is the Jordanian Anti-Doping Organization?	Yes	86	56	142	0.036	0.85	0.006	0.85
		15.6%	15.2%	15.5%				
	No	422	287	709				
		76.7%	77.8%	77.1%				
	Not sure	42	26	68				
		7.6%	7.0%	7.4%				
Using (or trading) doping is a crime punishable by law in Jordan.	Yes	251	148	399	2.747	0.097	0.055	0.097
		45.6%	40.1%	43.4%				
	No	60	75	135				
		10.9%	20.3%	14.7%				
	Not sure	239	146	385				
		43.5%	39.6%	41.9%				
There is a law related to doping in Jordan	Yes	255	152	407	2.393	0.122	0.051	0.122
		46.4%	41.2%	44.3%				
	No	46	49	95				
		8.4%	13.3%	10.3%				
	Not sure	249	168	417				
		45.3%	45.5%	45.4%				

**Table 3:** Perceptions about reasons for using the doping agents.

**Question**	**Answer**	**Pharmacist**	**Non** **HCP**	**Total**	**X^2^**	**P**	**Carmer V**	**P**
Bodybuilding (building muscle mass)	Neutral	47	46	93	22.25	<0.001	0.156	<0.001
		8.5%	12.5%	10.1%				
	Strongly disagree	7	8	15				
		1.3%	2.2%	1.6%				
	Disagree	32	43	75				
		5.8%	11.7%	8.2%				
	Strongly agree	297	150	447				
		54.0%	40.7%	48.6%				
	Agree	167	122	289				
		30.4%	33.1%	31.4%				
Maintaining weight	Neutral	169	124	293	1.347	0.853	0.038	0.853
		30.7%	33.6%	31.9%				
	Strongly disagree	13	11	24				
		2.4%	3.0%	2.6%				
	Disagree	149	93	242				
		27.1%	25.2%	26.3%				
	Strongly agree	69	45	114				
		12.5%	12.2%	12.4%				
	Agree	150	96	246				
		27.3%	26.0%	26.8%				
Improve physical performance	Neutral	44	44	88	8.947	0.062	0.099	0.062
		8.0%	11.9%	9.6%				
	Strongly disagree	6	8	14				
		1.1%	2.2%	1.5%				
	Disagree	32	31	63				
		5.8%	8.4%	6.9%				
	Strongly agree	271	167	438				
		49.3%	45.3%	47.7%				
	Agree	197	119	316				
		35.8%	32.2%	34.4%				
Improve mental performance	Neutral	165	116	281	4.312	0.365	0.068	0.365
		30.0%	31.4%	30.6%				
	Strongly disagree	23	20	43				
		4.2%	5.4%	4.7%				
	Disagree	173	101	274				
		31.5%	27.4%	29.8%				
	Strongly agree	82	68	150				
		14.9%	18.4%	16.3%				
	Agree	107	64	171				
		19.5%	17.3%	18.6%				
Imitating others	Neutral	80	68	148	26.363	<0.001	0.169	<0.001
		14.5%	18.4%	16.1%				
	Strongly disagree	14	19	33				
		2.5%	5.1%	3.6%				
	Disagree	54	65	119				
		9.8%	17.6%	12.9%				
	Strongly agree	203	126	329				
		36.9%	34.1%	35.8%				
	Agree	199	91	290				
		36.2%	24.7%	31.6%				
I support doping to be a legal substance in Jordan	Yes	51	49	100	3.655	0.056	0.063	0.056
		9.3%	13.3%	10.9%				
	No	447	280	727				
		81.3%	75.9%	79.1%				
	Not sure	52	40	92				
		9.5%	10.8%	10.0%				
I support the presence of an anti-doping authority in Jordan to reduce its consumption.	Yes	504	321	825	5.188	0.023	0.075	0.023
		91.6%	87.0%	89.8%				
	No	15	12	27				
		2.7%	3.3%	2.9%				
	Not sure	31	36	67				
		5.6%	9.8%	7.3%				

**Table 4:** Practicalities (prevalence and regimens used with the doping agents).

**Question**	**Answer**	Pharmacist	Non HCP	Total	X^2^	P	Carmer V	P
Have you ever taken a doping agent even if it was once?	Yes	20	36	56	14.453	<0.001	0.125	<0.001
		3.6%	9.8%	6.1%				
	No	526	330	856				
		95.6%	89.4%	93.1%				
	Not sure	4	3	7				
		0.7%	0.8%	0.8%				
Did you take a diet or medication after taking a doping agent to protect your body from its side effects? N=56	Yes	10	21	31	1.087	0.581	0.139	0.581
		50.0%	58.3%	55.4%				
	No	10	14	24				
		50.0%	38.9%	42.9%				
	Not sure	0	1	1				
		0.0%	2.8%	1.8%				
				31.9%				
I received information that these diets or drugs are good for the body. N=31	Yes	9	20	29	2.596	0.273	0.289	0.273
		90.0%	95.2%	93.5%				
	No	1	0	1				
		10.0%	0.0%	3.2%				
	Not sure	0	1	1				
		0.0%	4.8%	3.2%				
I think diet or medication helps the body to reduce the side effects of stimulant, N=31	Yes	9	19	28	0.764	0.682	0.157	0.682
		90.0%	90.5%	90.3%				
	No	1	1	2				
		10.0%	4.8%	6.5%				
	Not sure	0	1	1				
		0.0%	4.8%	3.2%				
I advise others to take these diets or (drug) N=31	Yes	9	15	24	1.827	0.401	0.243	0.401
		90.0%	71.4%	77.4%				
	No	0	3	3				
		0.0%	14.3%	9.7%				
	Not sure	1	3	4				
		10.0%	14.3%	12.9%				
The reason for taking these diets is to hide the presence of doping in the blood when performing a laboratory examination. N=31	Yes	2	5	7	0.570	0.752	0.136	0.752
		20.0%	23.8%	22.6%				
	No	7	12	19				
		70.0%	57.1%	61.3%				
	Not sure	1	4	5				
		10.0%	19.0%	16.1%				

**Table 5:** Practicalities for pharmacists as service providers.

**Question**	**Answer**	Females	Males	Total	X^2^	P	Carmer V	P
I was directly exposed to request a doping prescription or request help to get a doping agent	Yes	81	88	169	20.09	<0.001	0.191	<0.001
		23.8%	41.9%	30.7%				
	No	242	114	356				
		71.2%	54.3%	64.7%				
	Not sure	12	5	17				
		3.5%	2.4%	3.1%				
I was exposed to someone who asked me for an illegal doping agent	Yes	60	77	137	25.76	<0.001	0.216	<0.001
		17.6%	36.7%	24.9%				
	No	263	122	385				
		77.4%	58.1%	70.0%				
	Not sure	12	8	20				
		3.5%	3.8%	3.6%				
I feel responsible for educating people about doping prevention	Yes	302	177	479	5.596	0.133	0.101	0.133
		88.8%	84.3%	87.1%				
	No	14	19	33				
		4.1%	9.0%	6.0%				
	Not sure	19	11	30				
		5.6%	5.2%	5.5%				
I feel responsible for reporting doping dealers	Yes	240	133	373	6.759	0.08	0.111	0.08
		70.6%	63.3%	67.8%				
	No	43	44	87				
		12.6%	21.0%	15.8%				
	Not sure	52	30	82				
		15.3%	14.3%	14.9%				

## Data Availability

Data will be available upon request *via* e-mail to the corresponding author.
